# Effect of Gambisan on the Inhibition of Adipogenesis in 3T3-L1 Adipocytes

**DOI:** 10.1155/2013/789067

**Published:** 2013-08-20

**Authors:** Jung Won Kang, Dongwoo Nam, Kun Hyung Kim, Jeong-Eun Huh, Jae-Dong Lee

**Affiliations:** ^1^Department of Acupuncture and Moxibustion, College of Korean Medicine, Kyung Hee University, 1 Hoegi-dong, Dongdaemun-gu, Seoul 130-702, Republic of Korea; ^2^Oriental Medicine Research Center for Bone and Joint Disease, East-West Bone and Joint Research Institute, Kyung Hee University, 149 Sangil-dong, Gangdong-gu, Seoul 134-727, Republic of Korea

## Abstract

This study was conducted to explore the antiadipogenic effect and possible mechanism of Gambisan on 3T3-L1 cells. For quality control, Gambisan was standardized by HPLC and the standard compounds ephedrine, epigallocatechin-3-gallate, and caffeine were screened. Cultured 3T3-L1 cells that had been induced to differentiate were treated with various concentrations of Gambisan or its major component extracts (*Ephedra intermedia* Schrenk, *Atractylodes lancea* DC., and *Thea sinensis* L.) for 72 hours for MTT assay to determine cell viability or 10 days for LDH assay, triglyceride assay, DNA content measurement, Oil red O staining, RT-PCR, and western blot. Gambisan significantly inhibited adipogenesis in 3T3-L1 cells by reducing triglyceride contents and lipid accumulation in a dose-dependent manner without obvious cytotoxicity. Viability and DNA content in 3T3-L1 cells treated with Gambisan were significantly higher than cells treated with the major component extracts at every concentration. The anti-adipogenic effects of Gambisan appeared to be mediated by a significant downregulation of the expression of lipoprotein lipase mRNA and PPAR**γ**, C/EBP**α**, and SREBP-1 protein apart from the expression of hormone-sensitive lipase. Gambisan could act as a possible therapeutic agent for obesity. However, further studies including *in vivo* assays and clinical trials are needed to confirm the efficacy, safety and mechanisms of the antiobesity effects of Gambisan.

## 1. Introduction

Obesity, defined as an excessive body weight in the shape of fat accumulation that may impair health, is one of the most common global metabolic disorders, affecting up to an estimated 61% of adults including both overweight and obese. In particular, it has been conclusively linked with various medical conditions including dyslipidemia, diabetes mellitus, hypertension, some cancers, and osteoarthritis (OA) [1, 2].

 These relationships rely on the excessive differentiation and growth of adipocytes, the main constituent of fat, which leads to increased fat cell mass and number, adipogenesis including structural changes, lipid accumulation and lipogenic enzyme expression, and surplus energy accumulation stored as triglyceride (TG) in adipocytes [3–5]. Consequently, inhibition of the adipogenesis and TG deposition in 3T3-L1 adipocytes could be of crucial therapeutic importance in compounds developed to treat and even prevent obesity.

 The primary treatments of obesity are lifestyle modification, drug therapy, and bariatric surgery. The most basic and effective means of achieving productive lifestyle changes are a combination of behavioral therapies, dietary modification and regular exercise [6, 7]. However, pharmacotherapy is recommended when these kinds of approaches prove insufficient or when obesity is severe.

 Although some weight loss medications have been licensed and marketed in the past several, most including sibutramine, amphetamines, and rimonabant have been withdrawn because of serious adverse events including cardiovascular problems [8, 9]. There is a pressing need for developing antiobesity drugs that are efficacious and have minimal side effects.

 In this sense, there have been many studies exploring the effects and potential mechanism of candidate remedies from single medicinal herbs [10–12] or complex herbal prescriptions [13–16] because of their comparative safety and evidence identified by accumulated clinical experiences over hundreds of years in Asian countries including Korea.

 Gambisan, designated as HH911G in Kyung Hee University Korean Medicine Hospital (Seoul, Korea), is a novel antiobesity herbal extract formula composed of four medicinal herbs. It was formulated by modifying *Wolbigachultang* recorded in the traditional medical classic literature *Jinguiyaolue* and adding *Thea sinensis* L. to reinforce the weight-loss effect and to lessen the well-known side effects of its component herbs or standard compounds [17–22] through scholarly consensus among the faculty of College of Korean Medicine, Kyung Hee University. Despite the widespread clinical use of Gambisan, involving over 2,800,000 doses for the past 3 years, the mechanism of its therapeutic effect has not yet been elucidated.

 In the present study, the anti-obesity effect of Gambisan and its mechanism of action were investigated in differentiating mouse 3T3-L1 adipocytes [23], a basic and robust model to assess adipogenesis and adipocyte metabolism *in vitro*, by measuring viability, TG deposition, lipid droplet accumulation in the treated cells, and expression levels of several adipocyte-specific genes [24] including hormone-sensitive lipase (HSL) and lipoprotein lipase (LPL) and major adipogenic transcriptional factors [25] including peroxisome proliferator-activated receptor gamma (PPAR*γ*), CCAAT/enhancer binding protein alpha (C/EBP*α*), and sterol regulatory element binding protein-1 (SREBP-1).

## 2. Material and Methods

### 2.1. Materials

3-(4,5-Dimethylthiazol-2-yl)-2,5-diphenyl-tetrazolium bromide (MTT), Oil red O solution, 3-isobutyl-1-methylxanthine (IBMX), dimethyl sulfoxide (DMSO), dexamethasone (DEX), insulin (INS), isopropanol, formalin, and ethylenediaminetetraacetic acid (EDTA) were obtained from Sigma-Aldrich (St. Louis, MO, USA). Dulbecco's modified Eagle's medium (DMEM), fetal bovine serum (FBS), phosphate buffered saline (PBS), penicillin, streptomycin, and new bone calf serum (NBCS) were purchased from Gibco BRL (Grand Island, NY, USA).

 Trizol reagent was bought from Invitrogen (Carlsbad, CA, USA). Mouse monoclonal antibodies against PPAR*γ*, C/EBP*α*, and SREBP-1 were from Cell Signaling Technology (Beverly, MA, USA), and *β*-actin antibody was from Sigma-Aldrich. An reverse transcription-polymerase chain reaction (RT-PCR) kit and primers were provided by TaKaRa Biotechnology (Seoul, Korea). Horseradish peroxidase (HRP)-conjugated anti-mouse IgG antibody, cold lysis buffer, and 4–12% sodium dodecyl sulfate-polyacrylamide electrophoresis (SDS-PAGE) gels were acquired from Invitrogen.

 All other reagents, unless noted, were purchased from Sigma-Aldrich.

### 2.2. Preparation of Gambisan and Its Major Component Extracts

Gambisan is routinely mass produced in the Department of Pharmacy, Kyung Hee University Korean Medicine Hospital. Gambisan and its major component extracts (*Ephedra intermedia* Schrenk, *Atractylodes lancea* DC., and *Thea sinensis* L.) were obtained from Kyung Hee University Korean Medicine Hospital. They were identified by Dr. Nam-Jae Kim, director of the Department of Pharmacy, Kyung Hee University Korean Medicine Hospital. Voucher specimens were preserved at the Oriental Medicine Research Center for Bone and Joint Disease, Kyung Hee University. The constituents of Gambisan were *Ephedra intermedia* Schrenk (12 g), Gypsum Fibrosum (16 g),* Atractylodes lancea* DC. (8 g), and *Thea sinensis* L. (20 g).

 To prepare Gambisan, a ground mixture of *Ephedra intermedia* Schrenk, Gypsum Fibrosum, and* Atractylodes lancea* DC. was extracted twice at a respective ratio of 3 : 4 : 2 (w/w) with boiling distilled water for 2 hr in a reflux apparatus. The aqueous extracted solution was filtered and evaporated using a rotary evaporator (EYELA, Tokyo, Japan) *in vacuo* and lyophilized in a freeze dryer (EYELA) to completely remove residual solvent to produce a brown powder in a yield of about 19.44% compared with raw herbal materials. *Thea sinensis* L. alone was extracted separately as just described and Gambisan was finally manufactured by mixing the extracted mixture and *Thea sinensis* L. powdered extract at a ratio of 1 : 0.8.

 The major components of Gambisan were also extracted twice with boiling distilled water for 2 hr by reflux apparatus. The aqueous extraction was filtered and evaporated *in vacuo* and lyophilized in a freeze dryer to ensure complete removal of water yielding about 18.9%, 21.8%, and 31.5% of brown powder containing *Ephedra intermedia* Schrenk, *Atractylodes lancea* DC., and *Thea sinensis* L., respectively.

### 2.3. High Performance Liquid Chromatography (HPLC) Analysis of Gambisan and Its Standard Compounds

**Figure 1:**
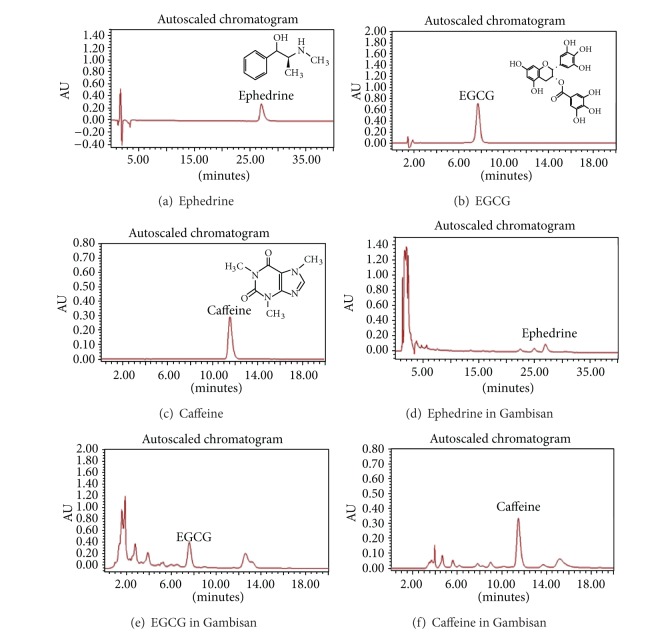
HPLC profiles of Gambisan and its standard compounds of ephedrine, EGCG, and caffeine. (a), (b), and (c) are profiles of standard ephedrine, EGCG, and caffeine, respectively. (d), (e), and (f) are profiles of ephedrine, EGCG, and caffeine extracted from Gambisan. Detection was done at 210 nm (about 27 min), 280 nm (about 7.5 min), and 280 nm (about 11.5 min), respectively.

Gambisan was standardized for quality control by HPLC analysis and the reference standard compounds (ephedrine, EGCG, and caffeine) were identified. Chromatographic analysis of Gambisan and its standard compounds was performed with a reverse-phase HPLC system (Waters, Milford, MA, USA) equipped with the Waters Breeze System (Alliance 2695 separation module and 2996 photodiode array detector (PDA)). Separation was carried out using a Hydrosphere C_18_ column (4.6 × 250 mm, particle diameter 5 *μ*m; YMC, Kyoto, Japan) at 30–45°C. The mobile phase of ephedrine, EGCG, and caffeine consisted of 0.1% phosphoric acid solution in sodium lauryl sulfate (SLS) : acetonitrile (can) : H_3_PO_4_ = 640 : 360 : 1, 20% tetrahydrofuran (THF), and SLS : ACN : H_3_PO_4_ = 900 : 100 : 1, respectively. Detection of ephedrine, caffeine, and EGCG was performed at 210 nm, 280 nm, and 280 nm, respectively (Figure 1).

### 2.4. Cell Culture, Differentiation, and Treatment

The 3T3-L1 mouse embryo fibroblasts were obtained from the American Type Culture Collection (ATCC CL-173; Manassas, VA, USA). Cells were cultured in high glucose DMEM supplemented with 10% NBCS, 100 U/mL penicillin, and 0.1 *μ*g/mL streptomycin to 90% confluence. The cells were induced to differentiate with differentiation medium (DMEM containing 5% FBS, 1 *μ*M DEX, 1 *μ*g/mL INS, and 0.5 mM IBMX). Beginning 2 days later, the medium was replenished with fresh differentiation medium every other day and maintained at 37°C in a humidified 5% CO_2_ atmosphere throughout the experiments until the cells were harvested. The differentiated 3T3-L1 cells were treated with various concentrations of Gambisan or its major component extracts. Cell viability after 72 hr was measured by MTT assay, and the cultured cells were used for the further analyses after 10 days.

### 2.5. Cell Viability Assay

#### 2.5.1. MTT Assay

The 3T3-L1 preadipocytes were seeded at a density of 1 × 10^4^ cells per well in 96-well plates and incubated in culture medium. The cells were then treated with various doses (0.01–1000 *μ*g/mL) of Gambisan or its major component extracts. After 72 hr, the cells were incubated in the dark with an MTT solution for 4 hr at 37°C. The supernatants were aspirated, DMSO was added to each well, and the plates were agitated to dissolve the formazan crystal product. Absorbance was then measured at 570 nm using a multiwell plate reader (Molecular Devices, Sunnyvale, CA, USA). The percentage of viable cells was calculated by defining the cell viability without treatment as 100%.

#### 2.5.2. LDH Assay

The 3T3-L1 preadipocytes were seeded at a density of 2 × 10^4^ cells per well in 24-well plates and cultured 90% confluence in culture medium. The cells were then treated with various concentrations (0.01–1000 *μ*g/mL) of Gambisan or its major component extracts for 10 days. As an indicator of cytotoxicity, the cytoplasmic enzyme lactate dehydrogenase (LDH) was measured in the culture supernatant of the Gambisan or its major component extracts-treated differentiating 3T3-L1 cells. An optimized LDH test (Promega, Madison, WI, USA) was used to quantify LDH activity in the medium of the treated differentiating 3T3-L1 cells. The percentage of viable cells was calculated by defining the cell viability without treatment as 100%.

### 2.6. TG Assay and DNA Contents Measurement

The cellular contents of TG were determined using a TG determination kit (Wako, Osaka, Japan) according to the manufacturer's instructions. Briefly, the cells were rinsed three times with PBS and scrapped off the plate with rubber policeman. The cells were extracted with 1 mL of lysis buffer (50 mM Tris, 0.15 M NaCl, 10 mM EDTA, and 0.1% Tween-20, pH 7.5 with HCl) followed by sonication for 30 sec at 4°C. Twenty microliters of the cellular lysate was mixed with 3 mL of the enzyme solution supplied and incubated for 10 min at 37°C. The absorbance at 550 nm was measured within 60 min. For internal control, the DNA concentration in 3T3-L1 cells was determined by spectrophotometry and DNA concentration in *μ*g/*μ*g was calculated.

### 2.7. Oil Red O Staining

The 3T3-L1 adipocytes were carefully washed twice with PBS and then fixed and dried with 10% formalin for 20 min. After removal of the formalin, 60% isopropanol was added to each well for 3 min and cells were washed thoroughly with PBS to remove unbound dye. Cells were then incubated with the Oil red O solution for 20 min and washed three times with distilled water. The staining of lipid droplets and cell morphology was ascertained microscopically and quantified by the i-solution program (IMT i-Solution, Seoul, Korea) using an Axiocam MRc5 CCD camera (Carl Zeiss, Jena, Germany) at ×200 magnification.

### 2.8. Gene Expression

Total cellular RNA was extracted from 3T3-L1 adipocytes using Trizol reagent and centrifuged at 12,000 rpm for 10 min at 4°C. One microgram of total RNA was reverse transcribed to cDNA for 60 min at 42°C and then 15 min at 72°C, using an RT-PCR system (TaKaRa Biotechnology, Seoul, Korea) that contained reverse transcriptase buffer, oligo (dT) 12-mer, 10 mM dNTP, 0.1 M dithiothreitol, reverse transcriptase, and RNase inhibitor. PCR using primers specific for each cDNA was carried out in a 20 *μ*L PCR reaction mixture supplied by TaKaRa supplemented with 2.5 units of TaKaRa Taq, 1.5 mM of each dNTP, 1x PCR buffer, and 20 pmol of each primer. The primer sequences of HSL, LPL, and *β*-actin used for gene expression analysis are shown in Table 1. The reaction was denatured at 95°C for 1 min, annealed at 55°C for 1 min, and extended at 72°C for 30 sec. Amplification for HSL or LPL was carried out using 30 cycles, and final extension was performed at 72°C for 7 min. An equal volume of each PCR was analyzed by 1.5% agarose gel electrophoresis and ethidium bromide staining. Signal intensity was quantified with the Gel Doc EQ (BIO-RAD Laboratories, Hercules, CA, USA). Marker gene expression was normalized to *β*-actin expression in each sample.

### 2.9. Western Blot Analysis

Following incubation, 3T3-L1 adipocytes were lysed with a ice-cold lysis buffer. Total proteins (20 *μ*g) were separated by 4%–12% SDS-PAGE under reducing conditions, and the separated proteins were transferred to Hybond-C nitrocellulose membranes (Amersham Biosciences, Piscataway, NJ, USA) at 300 mA for 90 min. After blocking with 5% nonfat skim milk for 2 hr, each membrane was probed with a 1 : 1,000 dilution of the particular primary antibody (mouse monoclonal antibody to PPAR*γ*, C/EBP*α*, SREBP-1, or *β*-actin) for 12 hr. The membrane was washed with Tris-buffered saline-Tween solution (140 mM NaCl_2_, 10 mM Tris-HCl, pH 8.0, and 0.05% Triton X-100) and incubated with a HRP-conjugated anti-mouse IgG antibody. Immunoreactive protein expression was visualized by an enhanced chemiluminescence (Amersham, Arlington Heights, IL, USA) and signal was detected using an Image Station 4000R (Kodak, New Haven, CT, USA). Anti-*β*-actinantibody was used to verify that equal amounts of proteins were loaded in all lanes.

### 2.10. Statistical Analysis

The values are expressed as the mean ± standard error of the mean (SEM). The data were analyzed by *t*-test or one-way analysis of variance (ANOVA) followed by Scheffe's multiple comparison test or Tukey's multiple comparison test for statistically significant differences between groups using the SAS statistical package (SAS Institute, Cary, NC, USA). Statistical significance was assessed at a level of *P* < 0.05.

## 3. Results

### 3.1. Effect of Gambisan and Its Major Component Extracts on Cell Viability in 3T3-L1 Cells

**Figure 2:**
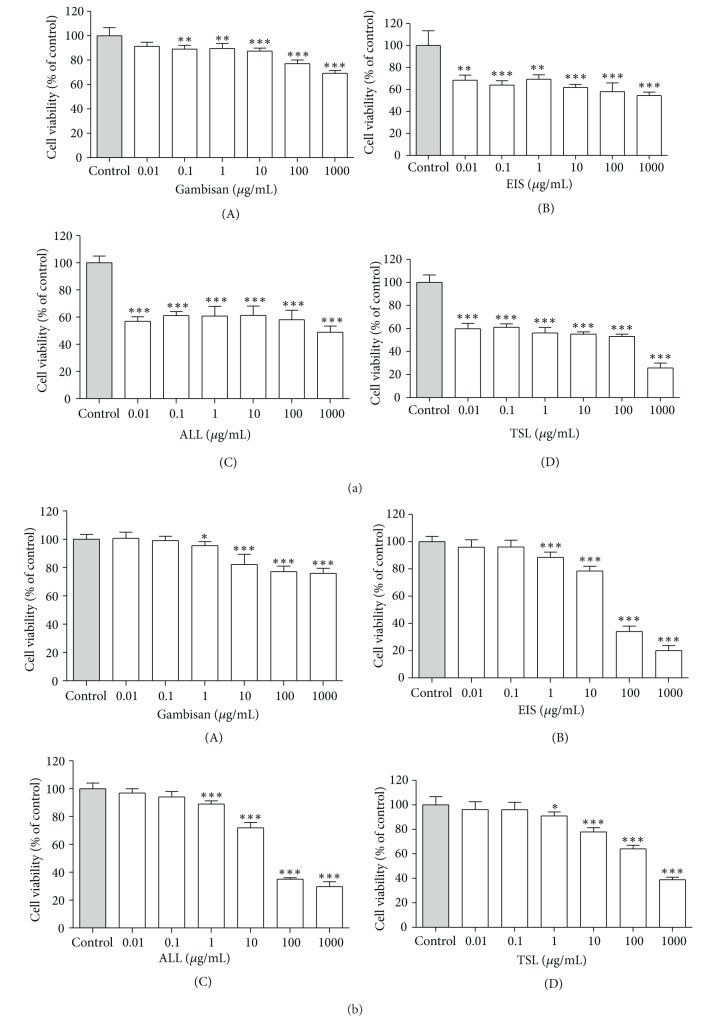
Effect of Gambisan and its major component extracts on 3T3-L1 cell viability. 3T3-L1 cells were treated with various doses (0.01–1000 *μ*g/mL) of Gambisan (A) or its major components (*Ephedra intermedia* Schrenk (B), *Atractylodes lancea* DC. (C) and *Thea sinensis* L. (D)). Cell viability was measured by the MTT assay after 72 hr (a) and by the LDH assay after 10 days (b). The percentage of viable cells was calculated by defining the cell viability without treatment as 100%. Values are expressed as mean ± SEM of six independent experiments. **P < 0.01, ***P < 0.001 compared with control (expressed by *t*-test). EIS: *Ephedra intermedia* Schrenk, ALL: *Atractylodes lancea* DC., TSL: *Thea sinensis* L., MTT assay: methyl thiazol tetrazolium assay, LDH assay: lactate dehydrogenase assay.

To examine the intracellular toxicity, 3T3-L1 cells were treated with a range of concentrations (0, 0.01, 0.1, 1, 10, 100, and 1,000 *μ*g/mL) of Gambisan and its major component extracts for 72 hr and 10 days, and the cell viability was determined using the MTT assay and LDH assay, respectively. Viability of 3T3-L1 cell populations treated with *Ephedra intermedia* Schrenk, *Atractylodes lancea* DC., and *Thea sinensis* L. for 72 hr was markedly decreased to the extent that the survival rates of cells were only about 60% even at the initial concentration of 0.01 *μ*g/mL (Figures 2(a)(B), 2(a)(C), and 2(a)(D)). However, Gambisan did not show a significant effect on 3T3-L1 cell viability at concentrations up to 100 *μ*g/mL, and the extent of inhibition did not exceed IC_50_ in all concentrations of Gambisan (Figures 2(a)(A) and 2(b)(A)). To focus specifically on the noncytotoxic range of Gambisan, concentrations of 0–500 *μ*g/mL were used in the following experiments. In addition, the viability in 3T3-L1 cells treated with Gambisan for 72 hr was significantly higher than that of populations treated with the major component extracts at every concentration (*P* < 0.0001, ANOVA with Scheffe's test), and the viability in Gambisan-treated 3T3-L1 cells for 10 days was also significantly higher than that of populations treated with the major component extracts at concentrations of 1, 10, 100 and 1,000 *μ*g/mL (*P* < 0.05, ANOVA with Tukey's test).

### 3.2. Effect of Gambisan and Its Major Component Extracts on TG Deposition and DNA Contents of 3T3-L1 Cells

**Figure 3:**
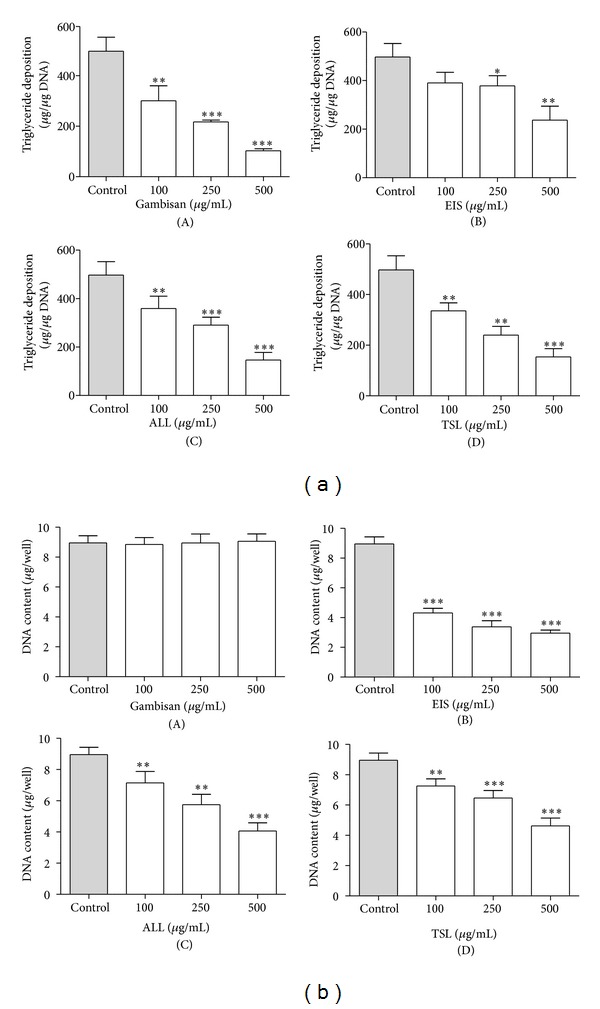
Effect of Gambisan and its major component extracts on the TG deposition and DNA contents in 3T3-L1 cells. 3T3-L1 cells were treated with various doses (0, 100, 250, and 500 *μ*g/mL) of Gambisan or its major components (*Ephedra intermedia* Schrenk, *Atractylodes lancea* DC., and *Thea sinensis* L.). After 10 days, the cellular TG contents (a) were measured with TG determination kit at 550 nm. The DNA contents in 3T3-L1 cells (b) were measured as internal control. Values are expressed as mean ± SEM of five independent experiments. *P < 0.05, **P < 0.01, ***P < 0.001 compared with control (expressed by *t*-test). EIS: *Ephedra intermedia* Schrenk, ALL: *Atractylodes lancea* DC., TSL: *Thea sinensis* L.

To evaluate the effect of Gambisan and its major component extracts on TG deposition and DNA contents of differentiating 3T3-L1 cells, 3T3-L1 cells were treated with various doses (0, 100, 250, and 500 *μ*g/mL) of Gambisan or its major components. After 10 days, TG and DNA contents in 3T3-L1 cells were determined. TG contents in 3T3-L1 cells treated with Gambisan and its major component extracts were significantly decreased in a dose-dependent manner. In addition, the TG contents in 3T3-L1 cells treated with Gambisan were significantly higher compared with its major component extracts at the concentrations of 150 and 500 *μ*g/mL (*P* < 0.0001, ANOVA with Scheffe's test) (Figure 3(a)). However, the DNA contents of 3T3-L1 cells treated with all concentrations (0, 100, 250, and 500 *μ*g/mL) of Gambisan were not significantly decreased, but the DNA contents of 3T3-L1 cells treated with its major component extracts were all significantly decreased in a concentration-dependent manner. The DNA contents of 3T3-L1 cells treated with Gambisan were significantly higher than its major component extracts at all concentrations (*P* < 0.0001, ANOVA with Scheffe's test).

### 3.3. Effect of Gambisan on Lipid Accumulation in 3T3-L1 Cells

**Figure 4:**
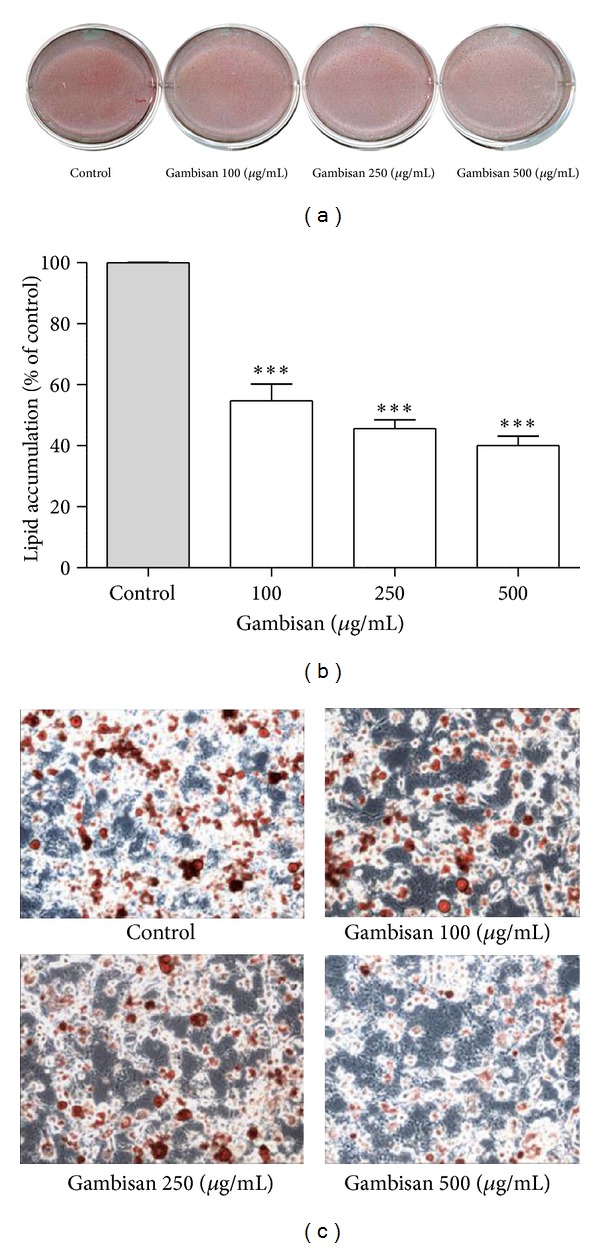
Effect of Gambisan on lipid accumulation in 3T3-L1 cells. 3T3-L1 cells were treated with various doses (0, 100, 250, and 500 *μ*g/mL) of Gambisan. After 10 days, cells were fixed and stained with Oil red O dye. Images of representative cells (a) scanned, (c) captured with a microscope (200x magnification), and (b) quantified by the i-solution program using a CCD camera are shown. The extent of lipid accumulation was expressed as the percentage of control. Values are expressed as mean ± SEM of five independent experiments. ***P < 0.001 compared with control (expressed by *t*-test).

To determine the effect of Gambisan on lipid accumulation in 3T3-L1 cells, 3T3-L1 cells were treated with various doses (0, 100, 250, and 500 *μ*g/mL) of Gambisan. After 10 days, intracellular lipid accumulation was examined with Oil red O staining for lipid droplets as an indicator of the degree of adipogenesis. As shown in Figure 4(c), copious Oil red O dye stained material accumulated in most of untreated control 3T3-L1 cells as evidence of adipogenesis. Microscopic images of treated 3T3-L1 cells captured after Oil red O staining revealed that the number of detectable stained droplets decreased as the concentration of Gambisan increased. In addition, the lipid accumulation rate was significantly reduced with all concentrations of Gambisan treatment compared with the control; the decrease was dose dependent (*P* < 0.001); accumulation was about 54.7%, 45.6%, and 40.0% at a Gambisan concentration of 100 *μ*g/mL, 250 *μ*g/mL, and 500 *μ*g/mL, respectively (Figure 4(b)).

### 3.4. Effect of Gambisan on Adipocyte-Specific Gene and Major Adipogenic Transcriptional Factor Expression in 3T3-L1 Cells

**Figure 5:**
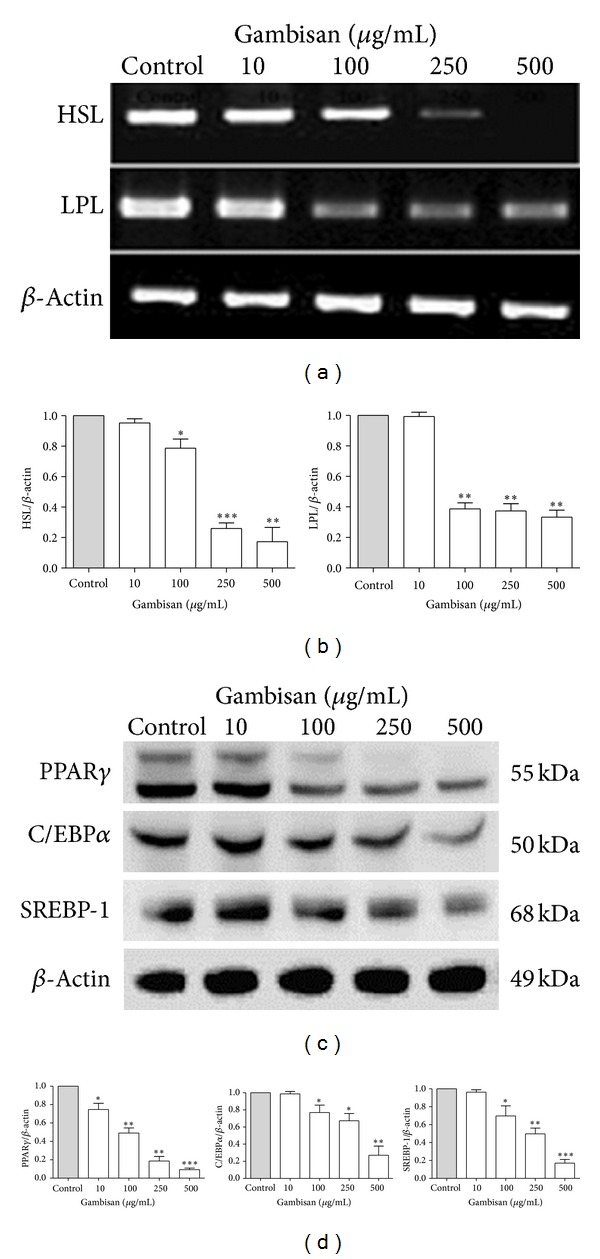
Effect of Gambisan on adipocyte-specific gene and major adipogenic transcriptional factor expression in 3T3-L1 cells. 3T3-L1 cells were treated with various doses (0, 10, 100, 250, and 500 *μ*g/mL) of Gambisan. After 10 days, the mRNA expression levels of HSL and LPL were measured using RT-PCR, and the protein expression levels of PPAR*γ*, C/EBP*α*, and SREBP-1 were measured using western blot analysis. Insets show representative RT-PCR band (a) and western blot analysis band (c) used for quantification, and bar graphs represent the band intensities of PCR products (b) and of western blots (d) adjusted by *β*-actin used as the internal control. Values are expressed as mean ± SEM of three independent experiments. *P < 0.05, **P < 0.01, ***P < 0.001 compared with control (expressed by *t*-test). HSL: hormone-sensitive lipase, LPL: lipoprotein lipase, RT-PCR: reverse transcription-polymerase chain reaction, PPAR*γ*: peroxisome proliferator-activated receptor gamma, C/EBP*α*: CCAAT/enhancer binding protein alpha, SREBP-1: sterol regulatory element binding protein-1.

To quantify the effect of Gambisan on adipocyte-specific gene and major adipogenic transcriptional factor expression in 3T3-L1 cells during adipogenesis, 3T3-L1 cells were treated with various doses (0, 10, 100, 250, and 500 *μ*g/mL) of Gambisan. After 10 days, RT-PCR and Western blot analysis were conducted. As shown in Figures 5(a) and 5(c), Gambisan reduced the mRNA expression of adipocyte-specific genes for HSL and LPL and the protein levels of major adipogenic transcriptional factors, such as PPAR*γ*, C/EBP*α*, and SREBP-1, in 3T3-L1 cells in a dose-dependent manner. In particular, all these expression levels were significantly reduced except HSL, LPL, C/EBP*α*, and SREBP-1 at the initial concentration of 10 *μ*g/mL of Gambisan compared with control (Figures 5(b) and 5(c)). These results were specific because *β*-actin levels were not affected.

## 4. Discussion

*In vitro* models have been meaningful in verifying the mechanisms related to the adipocyte differentiation, adipogenesis, and gene expression. Among others, 3T3-L1 preadipocytes, which can be induced to differentiate into adipocytes according to the coordinated program, are one of the most useful and established cell lines for researching the adipogenesis process [26, 27]. In 3T3-L1 cells, *in vivo* adipocyte development and lipid accumulation induction can be reliably reproduced equivalent to human preadipocytes [28]. For this reason, we naturally adopted these cells at the beginning of this research to identity the feasibility of Gambisan as a possible new anti-obesity herbal agent.

 The cell viability results of the present study imply that the decrease of TG deposition and lipid accumulation in 3T3-L1 cells treated with Gambisan, apart from its major component (*Ephedra intermedia* Schrenk, *Atractylodes lancea* DC., and *Thea sinensis* L.) extracts, was not due to its cytotoxicity, but to its own antiadipogenic properties. Meanwhile, it is true that herbal drugs are mostly prescribed as a compound formula of multiple medicinal herbs rather than a single herb, based on the combination principles of traditional Korean medicine [29]. Actually, the synergistic effects of mixed formula, usually termed as mutual reinforcement, may be induced by the interaction among herbs, and the adverse effects of any single component may be neutralized by another, a concept termed mutual suppression. So, we designed and conducted a cytotoxicity comparison experiment comparing Gambisan alone with its major component extracts. However, this issue still remains unsolved despite the interesting results that the cell viability and DNA contents in 3T3-L1 cells treated with Gambisan were significantly higher than its major component extracts at every concentration.

 The complex process of adipogenesis starts by the production of PPAR*γ*, which is controlled and activated by C/EBP*α* and SREBP-1. Among them, PPAR*γ* plays a role of a key transcription factor triggering and upregulating the subsequent expression of adipocyte-specific genes like adipocyte-fatty acid binding proteins (A-FABP) responsible for TG accumulation during adipocyte differentiation [30, 31]. C/EBP*α*, which is expressed rather late in the adipogenesis process, appears to promote differentiation in cooperation with PPAR*γ* by cross regulation [32]. SREBP-1 regulates lipogenic gene expression associated with fatty acid synthesis, which leads to increased synthesis of TG, and can contribute to the expression of PPAR*γ* ligands [33]. These adipogenic transcription factors act as master regulators of adipogenesis and lipid storage in 3T3-L1 cells. Because their overexpression can critically accelerate the differentiation of adipocytes, they may serve as potential therapeutic targets for obesity treatment and prevention. Considering the mechanism and western blot analysis results of these adipogenic transcription factors, they appear to be consistent with decrease in TG and lipid accumulation.

 HSL, which hydrolyzes triacylglycerol to monoacylglycerol and free fatty acids, is considered as one of the key lipolytic response genes related to lipid catabolism in adipocytes [34]. On the other hand, LPL is an early marker of adipocyte differentiation that dissolves lipid in lipoproteins into two free fatty acids and one monoacylglycerol to allow fatty acid entry into fat tissue, so LPL overexpression can indicate the initiation of lipid accumulation [35, 36]. In this study, the expression of both HSL and LPL was significantly inhibited by Gambisan treatment in a dose-dependent manner. In this sense, the anti-obesity effects of Gambisan appear to be exerted, not by the lipolysis pathway related with HSL, but by the dose-dependent downregulation of LPL.

 In conclusion, our results demonstrate that Gambisan efficiently inhibits adipogenesis in 3T3-L1 adipocytes as indicated by significant reduction in intracellular TG contents and lipid accumulation in a dose-dependent manner without eliciting apparent cytotoxicity. Furthermore, these suppressive effects of Gambisan are possibly mediated by down-regulated expressions of LPL mRNA and major adipogenic transcription activator (PPAR*γ*, C/EBP*α*, and SREBP-1) proteins of the adipogenesis pathway and via an HSL-independent pathway.

 Therefore, Gambisan may provide a possible therapeutic approach for the prevention and treatment of obesity. Further *in vivo* research and clinical trials are still needed to clarify the efficacy, safety, and precise molecular mechanisms of the anti-obesity effects of Gambisan.

## Figures and Tables

**Table 1:** Gene-specific forward and reverse primer sequences used for RT-PCR analysis.

Target gene names	Direction	Primer sequence	Annealing time (cycles)
HSL	Forward	5′-GAGGGACACACACACACCTG-3′	55°C (30)
	Reverse	5′-CCCTTTCGCAGCAACTTTAG-3′	

LPL	Forward	5′-AGTAGACTGGTTGTATCGGG-3′	55°C (30)
	Reverse	5′-AGCGTCATCAGGAGAAAGG-3′	

*β*-actin	Forward	5′-AGCCATGTACGTAGCCATCC-3′	55°C (32)
	Reverse	5′-TCCCTCTCAGCTGTGGTGGTGAA-3′	

HSL: hormone-sensitive lipase, LPL: lipoprotein lipase, RT-PCR: reverse transcription-polymerase chain reaction.
